# Understanding the complex systems driving the perceived obesogenic environment among adults in socioeconomically disadvantaged situations: Findings from a participatory Group Model Building process in Flanders

**DOI:** 10.1371/journal.pone.0346991

**Published:** 2026-10-05

**Authors:** Suzannah D’Hooghe, Yasemin Inaç, Sarah Dury, Benedicte Deforche, Karin De Ridder, Stefanie Vandevijvere, Delfien Van Dyck, Nico Van de Weghe, Leonor Guariguata

**Affiliations:** 1 Department of Epidemiology and Public Health, Sciensano, Leuven, Belgium; 2 Department of Public Health and Primary Care, Faculty of Medicine and Health Sciences, Universiteit Gent, Ghent, Belgium; 3 Department of Adult Educational Sciences, Faculty of Psychology and Educational Sciences, Vrije Universiteit Brussel (VUB), Brussels, Belgium; 4 Society and Ageing Research Lab (SARLab), Vrije Universiteit Brussel (VUB), Brussels, Belgium; 5 Department of Geography, Faculty of Sciences, Universiteit Gent, Ghent, Belgium; 6 Department of Movement and Sport Sciences, Faculty of Physical Education and Physiotherapy, Vrije Universiteit Brussel (VUB), Brussels, Belgium; 7 Department of Movement and Sports Sciences, Faculty of Medicine and Health Sciences, Universiteit Gent, Ghent, Belgium; 8 Access-To-Medicines Research Centre, Faculty of Economics and Business, KU Leuven, Leuven, Belgium; LSHTM: London School of Hygiene & Tropical Medicine, UNITED KINGDOM OF GREAT BRITAIN AND NORTHERN IRELAND

## Abstract

**Introduction:**

Persons in socioeconomically disadvantaged situations are disproportionally exposed to obesogenic environments and often have fewer resources to counteract their influence, resulting in poorer dietary outcomes and lower levels of physical activity. This study aimed to map the complex systems within the municipal obesogenic environment driving unhealthy eating behaviors and insufficient walking among adults in socioeconomically disadvantaged situations in Flanders, and to identify potential actions for local policymakers.

**Methods:**

Participatory group model building (GMB), a qualitative system dynamics method, was used to engage adults in socioeconomically disadvantaged situations (n = 7), local decision makers (n = 7) and local non-government stakeholders (n = 7) in two peri-urban Flemish municipalities. Two GMB sessions over two days were held in each municipality during which a causal loop diagram (CLD) was created and potential actions were identified for local policymakers by considering causal pathways and reinforcing loops in the system.

**Results:**

The CLD represents participants’ perceptions of how the local obesogenic environment contributes to unhealthy eating and insufficient walking among adults in socioeconomically disadvantaged situations. Six interconnected subsystems were identified: 1) social context, 2) facilities and activities, 3) mobility options, 4) information, 5) affordability, and 6) marketing and promotions. The perceived obesogenic environment, adverse health effects, and disadvantaged socioeconomic context in which individuals live mutually reinforce each other. Social and physical isolation emerged as important cross-cutting mechanisms, interacting with financial constraints, limited mobility, inadequate facilities, non-inclusive activities and restricted access to information.

**Conclusion:**

Participatory GMB and the resulting CLD provide a locally grounded basis for developing actions to promote healthy eating and walking among adults in socioeconomically disadvantaged situations. Actions addressing several subsystems simultaneously, while minimizing the effort required from residents to benefit from them, may be particularly valuable. Further co-design, feasibility assessment and sustained collaboration between residents, local stakeholders and decision-makers are needed to translate these findings into equitable local action.

## Introduction

Obesity, driven primarily by unhealthy eating and insufficient physical activity, is a complex, systemic and multi-causal issue [1]. Its prevalence worldwide is high and continues to increase, thereby contributing to an intensifying non-communicable diseases (NCD) epidemic [2]. In high-income countries, lower socioeconomic status is consistently associated with higher rates of overweight and obesity compared to a higher socioeconomic status, placing persons in socioeconomically disadvantaged situations at an elevated risk for NCDs and increasing health inequalities [3]. This pattern reflects both greater exposure to obesogenic environments and differential vulnerability, whereby fewer financial, time, mobility and psychosocial resources are available to buffer their influence [4].

It is increasingly recognized that both objective and perceived aspects of the physical activity and food environment influence eating behavior and physical activity [5–11]. Factors such as access to healthy foods, food prices, marketing, and social norms shape dietary behaviors, while walkability, transport infrastructure, availability and quality of recreational facilities, and perceived safety influence opportunities for physical activity [5,9,11–17]. Despite a substantial body of research describing the obesogenic environment and its dimensions, the prevention of obesity in community settings remains elusive [18]. Focusing on single environmental dimensions may miss the interactions between different factors influencing socioeconomic inequities in healthy eating behaviors and physical activity, and fails to incorporate dynamic elements, such as feedback and adaptations resulting from the interplay between people and their environments [19,20].

Systems thinking provides a valuable lens for studying such complexity. It allows researchers and stakeholders to conceptualize and visualize the dynamic relationships and feedback processes that reinforce obesogenic environments [21,22]. To date, most systems-based studies of obesogenic environments have been expert-led. A well-known example is the Foresight obesity map, developed for the UK government, in which experts mapped over one hundred interaction variables – clustered around a core energy-balance engine into domains such as food production, food consumption, individual psychology, physical activity and the built environment – to visualize why obesity is so resistant to single-focus interventions [23]. Other expert-led applications of systems thinking include systems map synthesized from literature reviews [10,19,24] and quantitative system dynamics and agent-based models [24–26]. These approaches have identified interacting macro-level drivers related to food production and marketing, the built environment, transport systems and socioeconomic conditions. Because such maps are primarily based on published evidence and expert knowledge, however, they may give less attention to how environments are perceived, navigated and experienced by the populations most affected.

Developing a system map with by people who know the system from within (e.g., local stakeholders, practitioners and community members) can situate the identified mechanisms within a specific decision-making context and promote shared understanding and ownership of both the model and its implications [27–29]. Studies comparing expert- and community-informed maps further suggest that participation influences the content of the resulting model. Community- and youth-led maps, for example, tend to place greater emphasis on social, environmental and psychosocial mechanisms, including stress, stigma and body image, whereas expert-developed maps give more prominence to physiological and individual-level determinants of the same problem [30–32].

Group Model Building (GMB) has emerged as a valuable participatory method within systems thinking, where a diversity of stakeholders participate in collaboratively conceptualizing causal loop diagrams (CLDs) to describe complex problems, generate dynamic hypotheses, and identify potential solutions [33]. GMB has its origins in operations research for optimizing industrial processes, but it is increasingly applied within health settings over the past decade. Studies have explored complex systems driving childhood obesity [22,28,34–36], unhealthy eating behavior [19,37,38] and insufficient physical activity [39]. These studies typically identify interrelated subsystems such as housing and built environment [12], transport infrastructure [12,26], health literacy [23], employment and work conditions [12,26], marketing and food promotion [23], and social norms [23,26]. Some of these studies extend beyond descriptive mapping by extracting key actions [28,37,39] or intervention development ideas [35].

Nevertheless, relatively few GMB studies have directly involved adults in socioeconomically disadvantaged situations in mapping the obesogenic environments that shape their everyday lives [40]. Consequently, limited evidence is available on how their perspectives influence the structure of system maps or the actions identified for them [41]. This restricts the development of community-based interventions that are both contextually appropriate and equity oriented [42,43].

Evidence is also limited for peri-urban settings, which combine urban and rural characteristics and remain underrepresented compared with urban environments. Such areas are becoming increasingly common across Europe and may face specific structural and mobility-related challenges, including limited transport, dispersed services and poorly accessible infrastructure [44–47].

The aims of this study were therefore (1) to jointly conceptualize the municipal obesogenic environment driving unhealthy eating and insufficient walking among adults in socioeconomically disadvantaged situations in two peri-urban Flemish municipalities through GMB, and (2) to explore actionable points for local governments to reverse current trends. Although walking, whether recreational or for transport, represents only one dimension of physical activity, it is an important component of everyday movement and strongly shaped by local environmental conditions [34].

The present study was conducted within the CIVISANO-project, a broader project that used mixed-methods to examine the role of objective and perceived environmental factors in physical activity and eating behavior among adults in socioeconomically disadvantaged situations in two peri-urban Flemish municipalities, Duffel and Herselt. The study protocol is described in detail elsewhere [48]. CIVISANO was conducted in close collaboration with local policymakers, stakeholders and residents, reflecting a shared concern about unhealthy eating and insufficient physical activity in the respective municipalities and the need for solutions targeting environmental determinants. Previous CIVISANO findings explored perceptions of adults in socioeconomically disadvantaged situations towards the local environment and its role in recreational walking behavior [49], walking for transport [50] and eating behavior [51]. GMB was used in the final phase of the project to integrate these findings with the perspectives of residents and municipal stakeholders and to identify locally relevant opportunities for action.

## Materials and methods

### Settings

The study took place in Duffel and Herselt, two medium-sized peri-urban municipalities in the province of Antwerp in the Flemish region of Belgium.

Duffel is a monocentric municipality geographically divided by a river, with most services, shops, and public facilities concentrated near the town center on the east side. It has a higher population density (796 inhabitants/km²) and includes several supermarkets and a train station, which provide relatively good access to services. However, green space is more limited and mainly concentrated along the river. Social housing is located both in and outside the center, making car or public transport use important for daily mobility. Duffel also has a large psychiatric hospital, and many former patients remain in the municipality through supported housing arrangements.

Herselt, in contrast, is more dispersed and consists of several sub-municipalities surrounded by wooded areas and agricultural land. It has a lower population density (283 inhabitants/km²), extensive green space, and multiple recreational outdoor areas, but fewer centrally located shops, fewer supermarkets, and no train station. Distances between facilities are longer, and public transport connections between sub-municipalities are limited. In addition to social housing, a notable feature of Herselt is the presence of low-income households living in campers that were originally intended as second residences. These housing situations are not fully regulated and often fall outside standard municipal housing frameworks.

Socioeconomic conditions also differ. Based on the deprivation index (percentage of children born in deprived households), Duffel (13.6%) is slightly above the Flemish average (≈12%), while Herselt (7.5%) is below it. Across both municipalities, adults in socioeconomically disadvantaged situations were mostly concentrated in social housing sites or dispersed rental apartments, often located outside central service areas, increasing reliance on mobility options to access food, activities, and public services.

### Ethical approval

This study was approved by the Medical Ethics Committee of Ghent University Hospital (BC-09260) and was conducted in line with the recommendations of the Belgian Data Protection Authority. All participants signed a written informed consent form prior to participation.

### Participant selection and recruitment

Local residents in socioeconomically disadvantaged situations and stakeholders from the local government and local non-government organizations were invited to participate during the period from June to August 2023. We critically reflected beforehand on who to invite for the group model building sessions, as we wanted to ensure an open and respectful space to exchange ideas and perceptions. Therefore, we invited adults in socioeconomically disadvantaged situations who previously participated in focus groups within the CIVISANO-project [49,52] and showed interest to exchange and discuss with local stakeholders. Participants in these earlier CIVISANO focus groups had been recruited through local intermediary organizations (e.g., social welfare services, poverty organizations and community centers), municipal channels, and snowball sampling, using the same socioeconomic eligibility criteria as described below [49,52]. For the GMB sessions, focus group participants who had expressed such interest were re-contacted by the research team. This prior participation familiarized them with the topics and with the experiences of other participants. In addition, previous findings were shared with stakeholders and policymakers involved in this study, ensuring that participants were informed about earlier results and pre-identified challenges faced by people in socioeconomically disadvantaged situations. The participants in socioeconomically disadvantaged situations had to be between 25 and 65 years old, reside in one of the involved municipalities and be considered socioeconomically disadvantaged by meeting at least two of the following criteria: no higher educational degree [53], no current paid or very low paid work [54], current perceived financial difficulties [55] and a perceived socioeconomic status of 5 or less on a 10-point scale (with 10 indicating the highest perceived SES and 1 indicating the lowest) [56]. In total, 4 adults in socioeconomically disadvantaged situations in Duffel and 3 adults in socioeconomically disadvantaged situations in Herselt agreed to participate. Among these 7 participants, there were four women (ages 31, 51, 56, and 63) and three men (ages 46, 56, and 62). In Duffel, 4 policy makers and 2 stakeholders from a local organization participated, and 3 policy makers and 5 stakeholders from local organizations participated in Herselt. In total, there were 10 participants in Duffel and 11 participants in Herselt, which corresponds with the recommendation to have at least 6 and at most 15 participants to conduct a group model building workshop [57]. The participant characteristics are shown in Table 1.

**Table 1:** Participant characteristics in the group model building workshops.

Municipality	Government	Local non-government organization	Citizens	Gender
Duffel	Health, welfare, safety, education, sport, youth, local economy, civil affairs, policy *n* = 4^*^	Poverty (*n* = 2)	Adults in socioeconomically disadvantaged situations (*n* = 4)	5 females 5 males
Herselt	Health, welfare, education, tourism, culture, communication, civil affairs, local economy, community work and participation *n* = 3^*^	Sports and free time (*n* = 1), social welfare (*n* = 2), poverty (*n* = 1), religion (*n* = 1)	Adults in socioeconomically disadvantaged situations (*n* = 3)	7 females 4 males

*The governmental stakeholders are responsible for several policy areas, e.g., one alderman is responsible for welfare, health, local economy and community work and participation.

### Group model building workshops

#### Workshop design and facilitation.

One two-session Group Model Building workshop was conducted in each municipality in August 2023. Each session lasted approximately three hours. The workshops aimed to develop a shared representation of the drivers and consequences of unhealthy eating and insufficient walking within the perceived municipal obesogenic environment and to generate potential actions for local policy makers.

The sessions were designed, organized and facilitated by the core modelling team, comprising the lead researcher (SD), a supporting researcher (YI) and an expert in GMB (LG).

The workshop design was informed by the Group Model Building principles and facilitation strategies outlined by Vennix et al. (1994), Andersen and Richardson (1997) and Luna-Reyes et al. (2006). Predefined but adaptable scripts were used to structure the sessions. All scripts can be accessed online from *Scriptapedia* (https://en.wikibooks.org/wiki/Scriptapedia). The sequence of workshop activities and scripts used are presented in Table 2.

**Table 2:** Workshop tasks and scripts used.

Session	Script name	Task description
**Day 1**	Introduction	Welcome, explaining objectives of the sessions, introducing the process of modelling, and briefly presenting the summarized results from the CIVISANO- project
	Hopes & fears	Sharing expectations regarding the GMB session and its outcomes
	Reference mode	Creating a mutual understanding and consensus of the dynamic and complex problem over time
	Variable elicitation (nominal group technique)	Identifying key variables related to ‘unhealthy environment’ with a focus on walking and eating behavior
	Creation of CLDs	Visualizing and linking variables, explaining causality and loops to create a CLD
	Feedback & evaluation	Evaluating and closing of the GMB session
**Day 2**	Introduction	Welcome, explaining objectives of this GMB session
	Model review	Summarizing dynamic insights, collective evaluation and capturing additional information on the CLD
	Action ideas (nominal group technique)	Identifying and prioritizing actions based on the CLD
	Feedback & next steps	Identifying next steps, evaluating, and closing of the GMB session

#### Problem framing and system boundary.

The first session began with an introduction to the objectives of the GMB sessions and the process of modeling. Findings from previous components of the CIVISANO-project were briefly presented to provide a shared starting point for the discussion.

Additionally, a ‘hopes & fears’ exercise took place in which participants could share expectations and express hopes and concerns regarding the GMB process and its potential outcomes (S1 Fig). In both workshops, participants were presented with a reference mode, as a simple graphical representation of the core problem over time (S2 Fig). This exercise supported the development of a shared understanding of the problem before variables and causal relationships were elicited.

The system boundary used for the GMB sessions was defined as the municipal environment, as perceived and experienced by adults in socioeconomically disadvantaged situations. Unhealthy eating and insufficient walking were defined as the two focal problem behaviors. Individual-level determinants and national or supranational policy levels were outside the primary scope of the exercise. However, participants could, and did, raise them when they considered them relevant because of how they operated through their local environment.

No predefined definition of “healthy” or “unhealthy” eating or walking was imposed. Participants were instead asked to discuss these behaviors according to their own understandings and experiences of practices that they believed supported or undermined health.

#### Variable elicitation and development of the municipal CLDs.

Once the problem was described and agreed on, participants were led in a variable elicitation exercise using the nominal group technique where they were asked to identify the main drivers and consequences within the perceived obesogenic environment of respectively unhealthy eating and insufficient walking in their municipalities (S3 Fig). Participants were encouraged to think in terms of causes and consequences and to explain how identified factors were connected.

The variables generated during the exercise were used to construct a causal loop diagram for each municipality. Variables were added to a large poster at the front of the room, after which participants identified causal directions and connections between them. The moderators supported the diagramming process by asking clarifying questions, helping participants articulate the direction of relationships and organizing the emerging structures visually. With support from the moderator, the variables and relationships were organized into causal pathways and feedback loops (S4 Fig).

Feedback loops operate between system variables. In reinforcing loops, a change in a variable propagates through the loop to amplify the initial change in the same direction, producing growth or decline; in balancing loops, the change propagates to counteract the initial change, as such an increase feeds back as a decrease and vice versa, giving the loop a stabilizing, goal-seeking character [43].

#### Digitization and participant review of the municipal CLDs.

After the first session, SD transferred the hand-drawn causal loop diagrams to a digital format using Kumu.io (2023). The moderator team used notes from the sessions to identify and name preliminary subsystems and feedback loops. The discussions were also audio-recorded, which allowed the research team to clarify and confirm notes where necessary.

During the second workshop, the digitized CLDs and the changes proposed by the moderator team were presented to participants for collective review (S5 Fig). Participants in both municipalities suggested edits and added some new variables (S6 Fig). The resulting municipal CLDs therefore reflected an iterative process involving participant elicitation, moderator-supported organization and subsequent participant review.

#### Generation of action ideas.

Throughout the process, the CLDs were treated as shared dynamic hypotheses about the factors and processes contributing to unhealthy eating and insufficient walking in the municipal environment. They were not considered empirically validated causal models. Identifying leverage points through a purely qualitative exercise is challenging and may lead to overinterpretation when a model has not undergone subsequent quantitative modelling or empirical testing.

Instead, participants used the causal pathways and feedback loops represented in the municipal CLDs as a basis for generating and prioritizing possible actions. This process followed a structured nominal group exercise based on the Scriptapedia “Action ideas” script. The exercise was intended to move from problem representation towards potential local responses while retaining a clear connection with the factors and relationships represented in the CLD. The resulting actions should therefore be interpreted as participant-generated, exploratory intervention ideas rather than as empirically validated leverage points.

#### Workshop evaluation.

At the end of the GMB sessions, all participants were invited to complete a brief, voluntary, and anonymous evaluation survey. Also, a short group discussion took place to revisit the hopes and fears identified at the beginning of the process and to reflect on whether participants’ initial expectations and concerns had been addressed.

#### Development of the merged summary CLD.

After the sessions in both municipalities, the modeling team integrated the final municipal CLDs into a single summary CLD. The purpose of this integration was not to compare Duffel and Herselt, but to synthesize the similarities between the two local models and create a more comprehensive representation of the shared system.

To maintain readability, the summary CLD only includes the causal connections required to represent the identified subsystems and named feedback loops. This approach is consistent with Finegood et al. (2010), who stated that by reducing the complexity of the visual image of the CLD, while preserving its underlying information, key relationships become more apparent [58].

The merged CLD was subsequently shared with participants for review, with the aim of determining whether it remained recognizable as a representation of the workshop discussions. No comments or requests for changes were received.

### Rigor and reflexivity

Rigor and traceability were supported through established Group Model Building practices, including predefined and adaptable Scriptapedia scripts, audio-recoding of the sessions, verification of the notes against recordings, documentation of the successive model versions (S5 and S6 Appendices in S5 and S6 Figs and the version history on Kumu.io), and participant review of the moderator-edited municipal CLDs and the merged CLD.

During manuscript revision, variable names and link polarities were also edited to improve consistency with causal loop diagramming guidelines. Specifically, directional wording was removed from variable names and corresponding polarity adjustments were made. Additionally, delay marks were added to relationships for which the modelling team considered that the effect would plausibly develop over time. These delays were identified through discussion within the research team and were not explicitly elicited or subsequently reviewed by participants; they should therefore be interpreted as modeler-added annotations. These editorial changes did not alter the causal structure previously reviewed by participants.

The core modelling team had established relationships with participants through previous CIVISANO focus groups. None lived in the participating municipalities, and their educational and socioeconomic positions differed from those of the participating residents. The team recognized that this positional distance, together with its environmental framing of obesity, could influence elicitation and interpretation. Participants’ own terminology was therefore retained where possible, no definitions of healthy or unhealthy behavior were imposed, and moderator interpretations were returned to participants for review. As in any qualitative inquiry, however, the subsequent processing and analysis of the workshop data — including the naming of subsystems and feedback loops and the synthesis of the two municipal CLDs — constituted necessary interpretive work by the research team, anchored in participants’ shared understanding through member checking and validation of the merged model.

## Results

Participants discussed and agreed that obesity-related behaviors, in particular unhealthy eating behavior and insufficient walking, have increased over time. Additionally, they agreed that the food and physical activity environments have become unhealthier over time, although they described this as more pronounced within the food environment. At the end of the GMB sessions, participants in Duffel produced two causal loop diagrams respectively one for the role of the perceived obesogenic environment in healthy eating behavior and one for recreational walking, while participants in Herselt chose to elaborate one causal loop diagram incorporating both behaviors (Appendix 5 and 6 in S5 and S6 Figs). The final summary CLD where all the overarching subsystems and feedback were combined is focusing on the perceived obesogenic environment that leads to both unhealthy eating behavior and a lack of walking presented in Fig 1. To enhance the clarity and readability of the diagram, not all connections between variables have been depicted. The full map is available online at Kumu.io.

**Fig 1:**
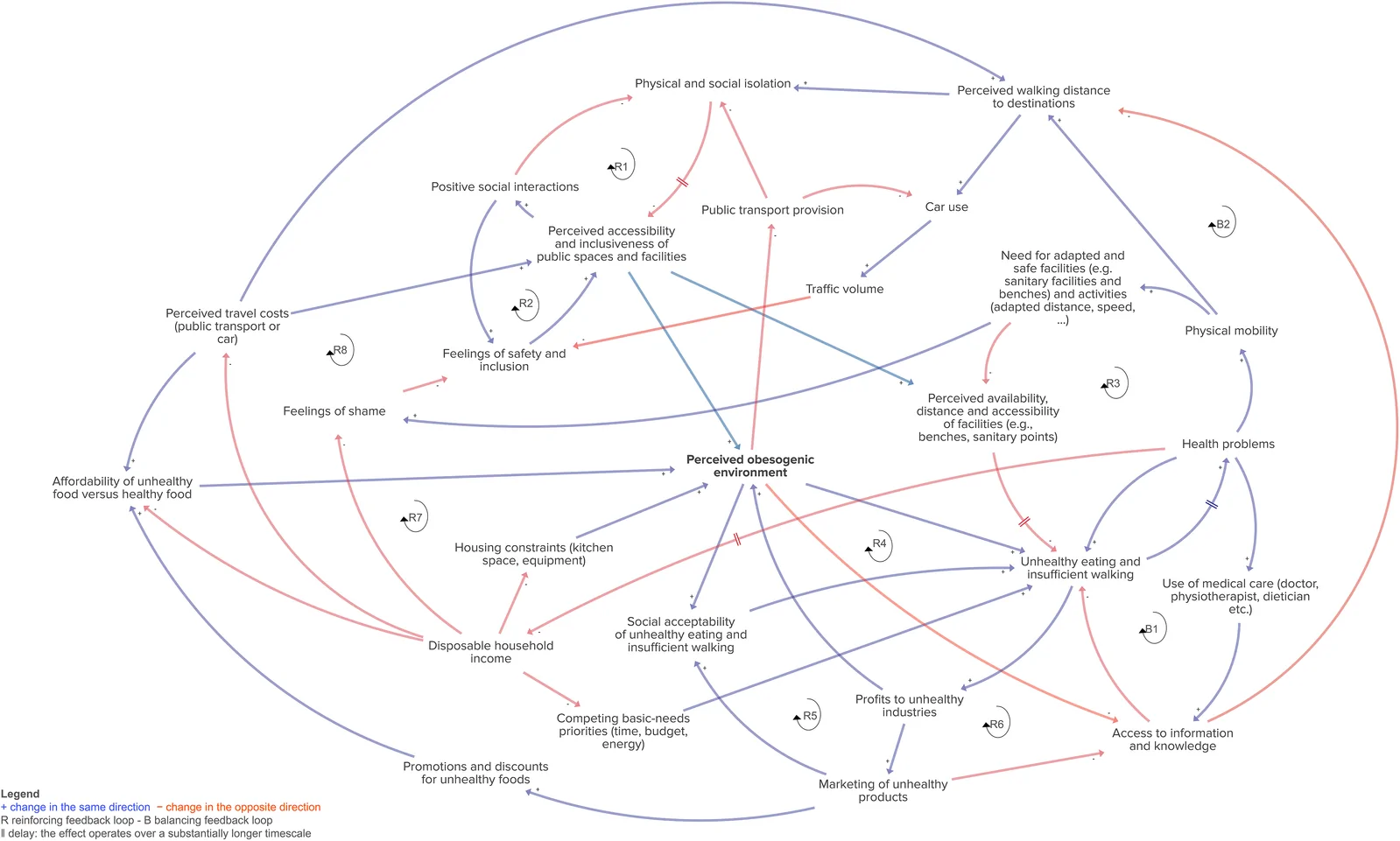
CLD for determinants of the perceived obesogenic environment among adults in socioeconomically disadvantaged situations. All labelled loops are described in full in Table 3.

Among our sample of participants in socioeconomically disadvantaged situations, the majority experienced health issues that exacerbated their socioeconomic disadvantage and financial hardship. Both the socioeconomically disadvantaged situation and the health problems negatively affected how they used and perceived their local environment. This environment was often perceived as obesogenic, contributing to unhealthy eating behaviors and reduced walking, which in turn worsened their health outcomes.

The following subsystems were identified by the researchers and agreed on by the participants: subsystem 1 – social context, subsystem 2 – facilities and activities, subsystem 3 – mobility options, subsystem 4 – information, subsystem 5 – affordability and subsystem 6 – marketing and promotions. Reinforcing loops (i.e., *if a variable increases, the effect through the cycle will return and increase the same variable*) are labeled with an ‘R’ and Balancing loops (i.e., *if a variable increases, the effect through the cycle will return and decrease the same variable*) with a ‘B’ [59]. An overview of all feedback loops identified in the CLD is provided in Table 3, including each loop’s type, the subsystems it spans, its full pathway with link polarities and delays, and its interpretation. In the subsystem descriptions below, loops are referenced by these labels (R1–R8, B1–B2).

**Table 3:** Feedback loops identified in the causal loop diagram, their type, the subsystems involved, and their interpretation. R = reinforcing loop; B = balancing loop; ‖ = the loop contains one or more delays. Variable names as in Fig 1.

Loop	Type	Subsystem(s)	Pathway in the CLD	Interpretation
**R1** Social connection–accessibility loop	Reinforcing	Social context	Positive social interactions → (−) physical and social isolation → (−) perceived accessibility and inclusiveness of public spaces and facilities → (+) positive social interactions	Everyday positive encounters draw people outside and reduce isolation, improving how accessible and inclusive public spaces feel; these spaces in turn serve as meeting spots that generate further encounters. The loop can spiral upward (connection) or downward (isolation).
**R2** Safety–social interaction loop	Reinforcing	Social context	Positive social interactions → (+) feelings of safety and inclusion → (+) perceived accessibility and inclusiveness of public spaces and facilities → (+) positive social interactions	Positive encounters increase perceived safety, which supports a positive perception of the social environment in which more encounters can take place; negative experiences (stigma, discrimination, public substance abuse) drive the same loop downward, discouraging walking.
**R3** Adapted facilities loop	Reinforcing	Facilities and activities	Health problems → (+) need for adapted and safe facilities and activities → (−) perceived availability, distance and accessibility of facilities → (−) unhealthy eating and insufficient walking → (+) health problems ‖	As health and mobility problems grow, so does the need for benches, sanitary points and adapted activities; where perceived availability falls short, people walk less, which over time worsens health and further raises the need.
**R4** Normalization–profits loop	Reinforcing	Marketing and promotion; social context	Obesogenic environment → (+) social acceptability of unhealthy eating → (+) unhealthy eating and insufficient walking → (+) profits to unhealthy industries → (+) obesogenic environment**/** → (+) marketing of unhealthy products → (+) social acceptability of unhealthy eating	Seeing others eat unhealthily normalizes the behavior, increasing its social acceptability and thereby the behavior itself, a dynamic that participants believed ultimately benefits the food industry through increased profits.
**R5** Marketing–affordability loop	Reinforcing	Marketing and promotion; affordability	Profits to unhealthy industries → (+) marketing of unhealthy products → (+) promotions and discounts for unhealthy foods → (+) affordability of unhealthy versus healthy food → (+) unhealthy eating and insufficient walking → (+) profits to unhealthy industries	Industry profits fund marketing strategies (discounts, larger package sizes) that make unhealthy food relatively more affordable, encouraging consumption and generating further profits.
**R6** Misinformation loop	Reinforcing	Marketing and promotion; information environment	Marketing of unhealthy products → (−) access to information and knowledge → (−) unhealthy eating and insufficient walking → (+) profits to unhealthy industries → (+) marketing of unhealthy products	Misleading promotions undermine people’s knowledge about healthy eating, counteracting the positive influence of accessible information and reinforcing unhealthy behavior, which sustains the profits that finance further marketing.
**R7** Poverty–health loop	Reinforcing	Affordability	Disposable household income → (−) affordability of unhealthy versus healthy food → (+) unhealthy eating and insufficient walking → (+) health problems ‖ → (−) disposable household income ‖	Lower income makes unhealthy options relatively more affordable, contributing to unhealthy behavior and, with delay, to health problems that make finding or maintaining employment harder — further reducing income. Health and employment effects operate over long timescales.
**R8** Mobility-poverty trap loop	Reinforcing	Affordability; mobility options; social context	Perceived travel costs → (+) perceived walking distance to destinations → (+) physical and social isolation → (−) perceived accessibility and inclusiveness of public spaces and facilities → (+) perceived availability, distance and accessibility of facilities → (−) unhealthy eating and insufficient walking → (+) health problems ‖ → (−) disposable household income ‖ → (−) perceived travel costs	Higher travel costs make distances feel longer and increase isolation, lowering the perceived accessibility and availability of facilities and reinforcing unhealthy eating and insufficient walking; over time, the resulting health problems reduce disposable income, which makes travel costs weigh even more heavily. Health and income effects operate over long timescales.
**B1** Medical care–information loop	Balancing	Information environment	Unhealthy eating and insufficient walking → (+) health problems ‖ → (+) use of medical care → (+) access to information and knowledge → (−) unhealthy eating and insufficient walking	People who develop health problems and seek medical help (doctor, dietician) gain access to information that can improve their knowledge and support behavior change, counteracting the initial deterioration.
**B2** Care–knowledge–accessibility loop	Balancing	Information environment; mobility options; social context	Unhealthy eating and insufficient walking → (+) health problems ‖ → (+) use of medical care → (+) access to information and knowledge → (−) perceived walking distance to destinations → (+) physical and social isolation → (−) perceived accessibility and inclusiveness of public spaces and facilities → (+) perceived availability, distance and accessibility of facilities → (−) unhealthy eating and insufficient walking	A larger balancing pathway across subsystems: knowledge gained through medical care reduces the perception that distances are too long to walk, lowering isolation and improving the perceived accessibility and availability of public spaces and facilities, which ultimately counteracts the initial rise in unhealthy eating and insufficient walking.

Loop pathways show only the variables constituting each loop; ‘+’ and ‘−’ denote change in the same and opposite direction, respectively.

A simplified version with the identified subsystems and how they interact is presented in Fig 2. This CLD reflects the joint discussion and perception of participants regarding how different elements of their environment contribute to an obesogenic environment. According to the participants, the obesogenic environment is influenced by, and in turn influences, all subsystems directly. Participants also described interconnections between subsystems. For example, they perceived the social context as being directly shaped by adverse health effects, a lack of transport options, low affordability, and a lack of adequate and accessible facilities and activities.

**Fig 2:**
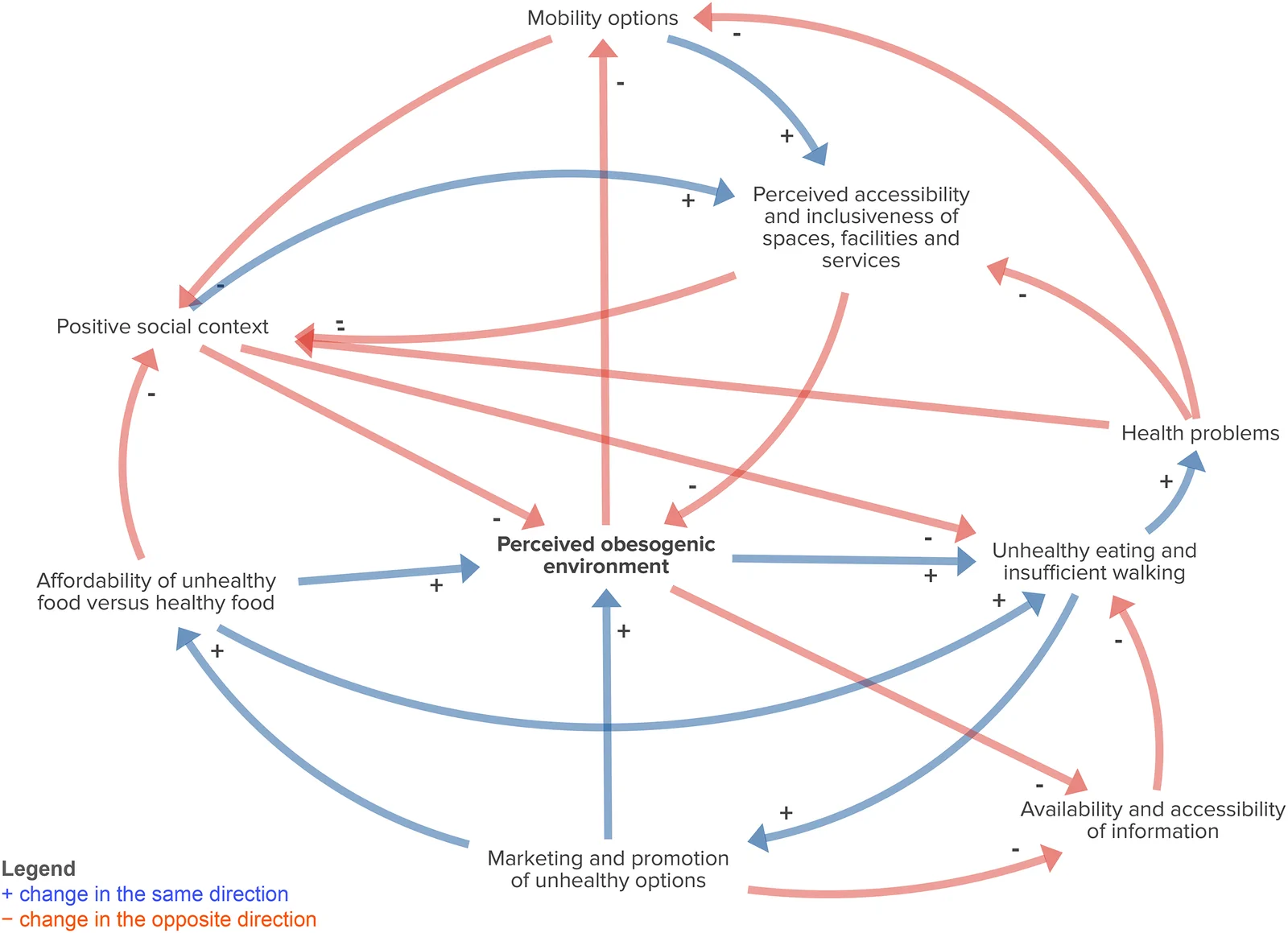
Simplified CLD for determinants of the perceived obesogenic environment among adults in socioeconomically disadvantaged situations showing feedback between subsystems.

Participants generated potential actions in groups using nominal group technique, informed by the causal pathways and feedback loops in the CLD, and exchanged ideas afterwards. The action ideas presented below (Tables 4–9) are participant-generated, exploratory intervention ideas. The level of change was determined by the aimed objective of the proposed action, which focused on either individual behavior change or community-level change (including physical, economic, sociocultural, and informational aspects). This categorization aligns with previous studies conducted in the context of the CIVISANO project, which utilized the Analysis Grid for Environments Linked to Obesity (ANGELO framework). Consequently, most participants were already familiar with these categories.

**Table 4:** Actions proposed by workshop participants and their level of change.

Subsystem 1: Social context		
Intervention point	Action	Level of change
Stimulating social interactions	Optimize public open spaces and create meeting places in different neighborhoods to stimulate social interactions	Community physical and social environment
	Organize local events in the neighborhoods to stimulate social interactions through support from the local government (both financially and through publicity)	Community social environment
	Create a ‘walk your neighbors’ dog’ campaign to stimulate social interactions in one’s street with a dog as an external motivation and a facilitator	Individual behavior change
	Organize local discussion groups to share information on a variety of themes of importance to the community members (e.g., local healthy food, leash off zones for dogs, etc.)	Individual behavior change
Fight against social isolation	Launch a service center with a variety of activities tailored to different needs, attracting different profiles	Community physical and social environment
	Do more organized and structural outreach work to identify and activate isolated people by actively informing and inviting them and organizing counselling at home	Individual behavior change
	Create opportunity where affordable and healthy hot meals can be home-delivered (already exists for older people in both municipalities, but to make this more widely available)	Community food environment
	Convert an old bus into an information point and low-cost food store that passes through sub-municipalities, where low-threshold encounters can be created	Community physical and social environment & individual behavior change
Improve social safety	Do not discriminate or stereotype in public communications	Community social environment
	Provide proper lighting on the streets (e.g., through movement sensors)	Community built environment
Improve physical safety	Create slow roads (limited number of cars and bicycles at low speed)	Community built environment
	Place signs with maximum speeds for cars and cyclists (for electric bikes and speed pedelecs)	Community built environment & Individual behavior change
	Enlarge walking and bike paths	Community built environment
	Increase awareness on consequences of high speed among cars and cyclists	Individual behavior change

**Table 5:** Actions proposed by workshop participants and their level of change.

Subsystem 2: Facilities and activities		
Intervention point	Action	Level of change
Create adequate facilities and inclusive activities	Launch a service center with a variety of activities tailored to different needs, attracting different profiles	Community physical environment
	Organize a variety of walking events, with the possibility of different distances and speed (e.g., a start-2-run or start-2-walk, but with more different levels and adapted to different needs)	Individual behavior change
	Organize adapted activities on a frequent base for people who are less mobile	Individual behavior change
	Create a working group to think and discuss actions to promote physical activity and healthy eating behavior (citizen participation)	Community environment
	Provide signposted routes, where distances and other facilities are indicated (e.g., availability of benches, sanitary facilities, road surface)	Community physical environment
Provide rest areas	Provide more benches in public open spaces	Community physical environment
	Work together with local stores to provide benches next to their doors (for example with a sticker saying that everyone is welcome to rest there)	Community physical environment
Provide sanitary facilities	Actively identify and maintain possible free public sanitary points (e.g., at the train station)	Community physical environment
	Create an accessible and easy-to-find website where all free sanitary points can be found	Community information environment
	Provide clear pictograms at public places, cafés, restaurants, stores etc. where people can use the sanitary facilities for free	Community information environment
	Create awareness around these pictograms and these places	Individual behavior change

**Table 6:** Actions proposed by workshop participants and their level of change.

Subsystem 3: Mobility options		
Intervention point	Action	Level of change
Improve proximity	Provide alternative transportation for the sub-municipalities to the local sport and service center (for older people or families with children without car)	Community physical environment
	Doing more outreach work (e.g., delivering groceries or affordable hot meals at home for those in need)	Individual behavior change
	Provide shared bicycles in both the center and the sub-municipalities	Individual behavior change
	Organize a healthy food market in each sub-municipality to increase proximity (e.g., with food surplus)	Community environment

**Table 7:** Actions proposed by workshop participants and their level of change.

Subsystem 4: Information environment		
Intervention point	Action	Level of change
Improve dissemination of information	Put activities that already exist in the communities in the spotlight. Provide practical and clear information and pay attention to visual attractiveness	Community information environment & Individual behavior change
	Devote more attention to search and implement effective communication strategies and use multiple communication channels to share the same message	Community information environment
	Work with pictograms to make information more accessible	Community information environment
	Make the municipality website more user-friendly and intuitive	Community digital information environment
	Provide LED screens with important information in strategic places	Community information environment
	Put more effort into information sharing with local organizations who can disseminate information to different audiences and target groups	Community information environment
	Convert an old bus into an information point that passes through sub-municipalities	Community information environment
Improve available information related to walking	Provide more signposted routes (with distances and other facilities such as benches, sanitary facilities and road surface indicated)	Community information environment
	Create a section in the municipality journal on walking activities	Individual behavior change
	Establish walking routes that pass local healthy merchants	Community information and food environment
Improve available information related to healthy eating	Organize workshops to learn and share how to cook healthy with limited ingredients and materials	Individual behavior change
	Create a local group (for example on Facebook or Hoplr) where more information can be found where to buy healthy and cheap products within the municipality, where discounts coupons can be shared, or where recipes can be shared	Individual behavior change
	Create an overview of all local stores and products on one website so that people can easily retrieve information on what is available and how to get there	Community information environment
	Provide more information on local farmers and products in the local newspaper	Community information environment
	Organize group visits to local farmers, with explanations on the fruits and vegetables available and how they can be used	Individual behavior change

**Table 8:** Actions proposed by workshop participants and their level of change.

Subsystem 5: Affordability		
Intervention point	Action	Level of change
Provide free or cheap (sport) equipment	Set up a lending service where free equipment can be used (e.g., walking shoes, bicycles, …)	Community environment & individual behavior change
	Organize a sports fair where cheaper equipment can be purchased	Individual behavior change
Provide flexible memberships	Provide sport facilities and parks or trails that are accessible at all hours of the day	Community environment change
	Provide walking groups or sport facilities where you can join without a fixed subscription or membership fee	Community environment & individual behavior change
Provide affordable alternatives for healthy eating	Create a social restaurant where people can eat a healthy meal for a low price	Community environment change
	Support local social organizations by facilitating collaborations with local businesses, supermarkets, and local farmers	Community environment change
	Organize a local food-surplus redistribution program within the municipality to connect local restaurants and stores that have surplus unsold food to adults in socioeconomically disadvantaged situations	Individual behavior change
	Set up Facebook groups where discount coupons can be shared or where group purchases can be made to keep the price down	Community environment change
	Organize food distributions more often but with smaller quantities so people don’t have to stockpile or freeze food at home	Community environment change
	Install community vegetable and herb gardens and picking gardens (not only in the center, but in all the sub-municipalities) for the neighborhood residents with information signs indicating positive properties of the herb or vegetable and how it can be used	Community food environment
	Provide free seeds, vegetable, and herb plants within the municipality to encourage use of vegetable or community gardens	Individual behavior change

**Table 9:** Actions proposed by workshop participants and their level of change.

Subsystem 6: Marketing and promotion		
Intervention point	Action	Level of change
Counter misinformation (un)healthy food	Make healthy food more attractive, through social media, in the municipality journal, as well as through social activities	Individual behavior change
	Organize visits to food stores and local farms highlighting and informing about healthy food	Individual behavior change
	Distribute more information about local healthy alternatives to the general supermarkets and fast-food take-outs	Individual behavior change

### Subsystem 1 – Social context

During the workshops, participants explained that poor health and related problems (such as chronic conditions, pain, or fatigue) are linked to mobility issues and a greater dependence on other people. These aspects were described as contributing to feelings of lower self-esteem and shame. According to the participants, such feelings discourage social interactions and therefore increase social isolation. Several participants emphasized that this social isolation is not only problematic in itself, but also because it makes asking for help more difficult. As they explained, being isolated and dependent on others can increase the threshold to reach out, whether for practical support or to access public centers such as social welfare services or local food distributions. This, in turn, reinforced the negative perception that public spaces and facilities are inaccessible or not inclusive, which further discourages walking and healthy eating. Some participants also shared that a lower self-esteem and shame often directly trigger unhealthy eating behavior, such as eating for comfort or to reduce stress. At the same time, participants stated that small, everyday positive social encounters (for example greeting neighbors or chatting with passersby) leads to an increased motivation to get outside and walk, reducing the social isolation and ameliorating the perception of accessibility and inclusiveness of public spaces and facilities (e.g., parks or public open spaces) (R1). They also highlighted that these spaces could serve as meeting spots, further promoting such positive interactions (R1). Participants stressed that these positive encounters increase their feelings of safety, while negative social interactions (such as experiencing stigma or discrimination) or public drug and alcohol abuse evoke fear or feelings of threat. These negative experiences reinforced their perception of public spaces as unsafe, which discouraged walking (R2). Conversely, a perceived sense of safety will also reinforce the positive perception of the social environment, as more encounters can take place in a safe manner (R2). Lastly, participants stated that watching people’s unhealthy eating habits in their surroundings and observing more and more unhealthy food around them seem to normalize this. This, in their perception, created a kind of social acceptance, which in turn reinforced unhealthy eating behaviors, the same reinforcing dynamic the participants linked to industry profits (R4).

#### Areas for action.

Stimulating social interactions, fighting against social isolation, and increasing the feelings of physical and social safety were identified by the participants as potential interventions points to promote walking and healthy eating behavior (Table 4).

### Subsystem 2 – Facilities and activities

According to the participants, persons with health and mobility issues have an increased need for adequate and safe facilities, such as the sufficient presence of sanitary facilities or benches to rest. They described how the fear that these facilities are not present or within reach contributes to people walking less (R3). Additionally, participants also perceived that persons with health and mobility issues are more likely to need activities that are adapted to their conditions (e.g., a walking group that organizes activities with varied speeds and distance). The perceived inappropriateness of facilities and activities was mentioned by participants in socioeconomically disadvantaged situations as a barrier because of feelings of shame about one’s condition, which participants felt reinforced physical and social isolation, feeding back into the social-context dynamics, described in Subsystem 1 (R1, R2).

In combination with limited mobility options (e.g., no car or bicycle ownership or insufficient public transportation), participants expressed that these factors contribute not only to a perceived lack of accessibility of public spaces and facilities, but also to actual structural constraints that limit where people go and what they can access. This in turn discourages walking and supports unhealthy eating.

#### Areas for action.

The creation of adequate and inclusive activities, with the provision of rest areas and sanitary facilities, was identified as intervention points by the participants (Table 5).

### Subsystem 3 – Mobility options

Participants stated that people who experience mobility issues may perceive distances as too long to cover on foot. They described how this perception contributes to physical and social isolation, lowering the perceived accessibility of (healthy) food stores, facilities, or public spaces, and thereby reinforcing the perceived obesogenic environment and therefore unhealthy eating and insufficient walking. This chain constitutes a part of the reinforcing loop R8, described in full in Subsystem 5.

At the same time, participants emphasized that this is not only a matter of perception. They pointed out concrete structural barriers, such as limited car and bike ownership due to low affordability and inadequacies in public transport in terms of the areas served and the time schedule. Additionally, participants explained that their dependence on limited public transport offerings further reinforced physical and social isolation. These limited mobility options were described as reducing opportunities to use diverse facilities (such as stores, local community centers and parks) that are not in their immediate vicinity, which participants felt further reinforced insufficient walking and unhealthy eating.

#### Areas for action.

To improve the mobility options and perceived proximity was identified as an intervention point for this subsystem by the participants (Table 6).

### Subsystem 4 – Information environment

Participants stated that a lack of awareness of walking trails or attractive walking facilities or public places in the neighborhood contributes to insufficient walking. They pointed out that this is not merely a matter of residents’ awareness, but also pertains to the availability and accessibility of information within the municipality regarding existing facilities and walking activities. Additionally, they mentioned that insufficient walking will also contribute to this lack of awareness, as people won’t go out and therefore do not ‘discover’ their neighborhood. Healthy eating behavior also involves the availability and accessibility of information on healthy foods, where it can be found and how it can be used. According to our participants this will promote healthy eating behavior. Participants assumed that persons with health problems who seek and receive medical help from a doctor or dietician have access to this information and can thus increase their knowledge and change their health behavior (B1). A related, larger balancing loop operates across several subsystems (B2). As unhealthy eating and insufficient walking increase, health problems accumulate (with delay), leading more residents to use medical care and thereby gain access to information and knowledge. This improved knowledge reduces the perceived walking distance to destinations, which lowers physical and social isolation, improves the perceived accessibility of public spaces and the perceived availability of facilities, and ultimately counteracts the initial rise in unhealthy eating and insufficient walking. Furthermore, it was also cited that while community members know the major supermarkets in the municipality, there is very little awareness of and familiarity with (healthy) local alternatives (e.g., local farmers, community gardens or picking fields), which also reinforces unhealthy eating.

#### Areas for action.

Providing accessible information and disseminating and communicating about this in an effective way were identified as key intervention points within this subsystem (Table 7).

### Subsystem 5 – Affordability

Participants in socioeconomically disadvantaged situations emphasized that a disadvantaged socioeconomic situation of community members directly reduced both their actual and perceived ability to afford healthy options. They explained that this lack of ability to afford healthy options contributed to unhealthy behaviors, such as frequent consumption of unhealthy food or insufficient physical activity, which were perceived to contribute to poor health. In turn, poor health was described as making it harder to find or maintain employment, which reinforced a disadvantaged socioeconomic situation, creating a reinforcing feedback loop (R7) in which lower disposable income, reduced affordability of healthy options, unhealthy behaviors and health problems perpetuate one another over time. The health and employment effects in this loop operate with considerable delay.

They noted that promotions and discounts were more common for unhealthy food. In addition, participants explained that people with specific dietary needs (e.g., sugar-free, lactose-free or vegan products) often had to purchase more expensive alternatives, which they felt contributed to the perception that healthy eating is less affordable.

Costs related to transportation were also highlighted. Participants perceived that travel costs, such as fuel or mileage costs for cars and the price of public transportation, limited their access to mobility. Tracing this link further, reveals that it is embedded in a larger reinforcing loop (R8). Higher travel costs increased perceived walking distances, creating barriers to reaching facilities outside their immediate environment. As a result, they felt discouraged from going out, which reinforced physical and social isolation and limited opportunities for walking and healthy eating. Over time, the resulting health problems reduce disposable household income, which in turn makes travel costs weigh more heavily.

Additionally, the participants linked a limited budget and a lack of disposable income to living in social housing with often unfavorable housing conditions such as restricted kitchen space (e.g., limited storage space or lack of a freezer) and limited cooking equipment. They explained that this made home-cooking more difficult and reduced the affordability of buying larger or frozen quantities, which were perceived to be cheaper.

Finally, participants described how financial constraints also influenced ‘time and mental affordability’. They explained that work or household responsibilities took priority, and there was less energy or time available for walking or home-cooking. Moreover, they stated that a limited budget makes it more difficult to buy appropriate walking equipment (e.g., hiking shoes or rain equipment), which they felt reduced the ability to engage in walking. Additionally, participants mentioned that the presence of unhealthy prepared products (such as fast food or ready-to-eat meals in stores) in combination with limited time, other priorities and insufficient cooking equipment contributed to unhealthy eating behavior.

#### Areas for action.

The provision of affordable equipment and alternatives for healthy eating behavior were identified as important intervention points (Table 8).

### Subsystem 6 – Marketing and promotion

Participants mainly referred to the role of the food industry and advertising in relation to eating behaviors. They explained that an increasingly obesogenic environment, in which unhealthy food is omnipresent and visibly consumed, raises the social acceptability of unhealthy eating, which in turn reinforces unhealthy eating and insufficient walking. Participants believed that this ultimately benefits the food industry through increased profits, and that these profits in turn stimulate the industry to further shape the obesogenic environment, closing a reinforcing feedback loop (R4). In addition, according to the participants, the industry reinvests these profits in marketing strategies, such as offering more discounts on unhealthy food and promoting larger package sizes. They felt that these practices make unhealthy food appear more affordable compared to healthier options and further increase the social acceptability of unhealthy eating, encouraging overconsumption (R5). Participants also expressed concerns that such marketing strategies can mislead consumers. For example, they described how promotions may give you the impression that products are cheaper, even when this is not the case. In their views, these tactics contribute to misinformation that undermines people’s knowledge about healthy eating. They explained that this misinformation can counteract the positive influence of accessible information in the environment, ultimately reinforcing unhealthy eating behaviors (R6).

#### Areas for action.

Notably, all actions proposed within this subsystem aim to make healthy food more attractive or more visible (Table 9). No participant proposed limiting marketing practices they had themselves described as harmful.

## Discussion

This study used Group Model Building to explore adults in socioeconomically disadvantaged situations, local policymakers and community stakeholders perceived the municipal systems contributing to unhealthy eating and insufficient walking in two peri-urban Flemish municipalities. Six closely connected subsystems were identified: 1) social context, 2) facilities and activities, 3) mobility options, 4) affordability, 5) information, and 6) marketing and promotion. The main contribution of the study lies not in identifying these determinants individually, many of which are already well documented, but in showing how they interact and accumulate in everyday life. In particular, limited mobility, financial constraints, restricted access to information and social isolation were perceived to reinforce one another, reducing residents’ opportunities to access healthy food, participate in activities and maintain social connections.

### Recurrent and context-specific system dynamics

Several of the identified mechanisms are consistent with previous systems maps of obesity, dietary behavior and physical activity. These include reinforcing relationships between socioeconomic disadvantage, stress and poor health; the interaction between food affordability, exposure to unhealthy food and dietary preferences; the influence of social norms and marketing; and the way transport and the built environment shape access to food and opportunities for physical activity [10,60–63]. An expert-developed map of obesity in socioeconomically disadvantaged urban neighborhoods in the Netherlands similarly identified mutually reinforcing dynamics across the food, physical activity, socioeconomic and sociopolitical environments. It highlighted, among other mechanisms, the interaction between unhealthy food supply and demand, chronic stress, income insecurity, limited facilities and institutional distance [62]. A recent participatory study among people living on a low income also identified scarcity and wellbeing, the unhealthy food environment, and social and societal barriers as three central and interacting dynamics shaping dietary behavior [64].

The similarity between the municipal CLDs also suggests that some dynamics extend beyond the specific characteristics of Duffel and Herselt. This is consistent with Calancie et al., who compared participatory CLDs developed in seven communities across the United States and identified recurring dynamics related to intergenerational poverty, difficulty meeting basic needs, stress and mental health, healthy food supply and demand, safety, social norms and community engagement [35]. Such convergence does not imply that local context is unimportant. Rather, it suggests that common mechanisms may be expressed differently depending on the spatial, institutional and socioeconomic characteristics of a community.

Within the present study, mobility appeared to be a particularly important cross-cutting mechanism. In the peri-urban municipalities studied, dispersed settlement patterns, longer distances, limited public transport and the location of some social housing outside central service areas affected more than transport alone. Mobility shaped access to affordable food, recreational opportunities, public services, information and social networks. It therefore functioned as a gateway connecting several subsystems. These findings extend the concept of mobility poverty by illustrating how transport disadvantage can compound other forms of socioeconomic disadvantage and contribute simultaneously to unhealthy eating, insufficient walking and social isolation [47,65].

The centrality of social isolation is another notable feature of the resulting model. Previous studies have often emphasized the protective role of social support, family modelling and community organizations [13,34]. Participants in this study instead described how isolation could emerge from the interaction of inaccessible transport, physically dispersed housing, limited information, financial constraints, stigma and a shortage of inclusive opportunities for social participation. Social isolation was therefore not represented solely as an interpersonal problem but as an outcome of the organization of the wider local environment. It also fed back into other subsystems by reducing opportunities to exchange information, participate in activities, seek support and build the confidence needed to use available services.

### Implications for action across subsystems

From a systems perspective, actions addressing several subsystems simultaneously may offer greater potential than isolated interventions. Four participant-generated actions were particularly cross-cutting: a multifunctional service center offering tailored activities; a mobile service combining an information point with access to affordable food across the sub-municipalities; clearly signposted walking routes with information on distances, benches and sanitary facilities; and home delivery of affordable healthy meals combined with proactive outreach.

These actions could generate multiple, mutually reinforcing benefits. For example, a mobile food and information service could simultaneously reduce transport barriers, improve access to affordable food, provide information and create opportunities for social contact. Similarly, a multifunctional service center could combine practical support, inclusive activities and opportunities to strengthen social networks. These actions should not yet be interpreted as formally identified leverage points, as the study did not assess their likely effect sizes, feasibility, costs or unintended consequences. They nevertheless represent promising candidates for further co-design and feasibility testing because they respond to several of the barriers represented in the CLD rather than addressing one factor in isolation.

The findings also illustrate the relevance of proportional universalism. Community-level improvements may benefit the population generally, but additional support is likely to be necessary for residents experiencing overlapping financial, mobility, health and social barriers [66]. The Depth Framework further distinguishes interventions according to their exposure, mechanism and level of engagement [67]. Many actions proposed in the workshops would improve the environment but would still require residents to identify, understand and actively use the available support. Their effectiveness may consequently remain dependent on individual time, confidence, health, mobility and cognitive capacity.

To reduce these agentic demands, environmental improvements could be combined with proactive and tailored forms of outreach. For example, providing information about existing activities may have limited effect when residents are socially isolated, lack transport or are uncertain whether they will feel welcome. Combining accessible activities with transport support, personal outreach and affordable participation could address these barriers together. Similarly, increasing the availability of healthy food is unlikely to be sufficient when affordability, transport and opportunities for food preparation remain constrained. Coordinated actions across policy domains are therefore needed rather than a collection of disconnected initiatives.

### Interpretation of the feedback structure

Participants initially identified one balancing loop, in which people experiencing health problems could obtain professional guidance, increase their knowledge and make changes that support healthier behavior. One additional balancing dynamics were identified when the diagram was reviewed during manuscript revision. The predominance of reinforcing loops may have several, non-exclusive explanations. First, it may represent the underlying system structure: a local environment that largely promotes unhealthy eating behaviors and insufficient walking, and therefore requires deliberate action to shift existing dynamics. Second, the design and focus of the elicitation process may have influenced the types of feedback dynamics identified. The sessions were primarily oriented towards eliciting participants’ perceptions and causal narratives, rather than systematically probing for system limits or balancing mechanisms. Third, modelling and naming decisions can influence the apparent polarity of a loop. Directionally phrased variables may lead a balancing relationship to be represented as a sequence of positive causal links within an apparently reinforcing narrative. Finally, the small number of balancing loops should therefore not be interpreted as evidence that no stabilizing mechanisms were present in the local system. The model remains a simplification representation of perceived causal relationships, based on participants’ perceptions and may therefore omit relevant mechanisms or represent them imperfectly.

### Limited representation of political and environmental change

No variables explicitly represented local political decision-making, policy investment or institutional accountability, despite the participation of policymakers in the sessions. Moreover, many proposed actions focused on information, awareness or individual support rather than on changing upstream environmental conditions. Similar tendencies have been observed in other participatory studies [28,68]. One explanation for this tendency could be that individuals, whether residents or policymakers, are accustomed to framing obesity in terms of personal responsibility, which is a dominant narrative in public health discourse [69,70]. Previous research shows that systemic and upstream determinants are often less visible and more difficult for laypeople and local stakeholders to conceptualize compared to concrete, individual-level solutions [71]. For local policymakers, interventions targeting individual behaviors are often seen as more straightforward, requiring fewer resources and shorter implementation timelines, whereas systemic changes demand significant infrastructure investment and longer-term commitment [72]. This may also be reinforced by limited budgets and rigid decision-making structures, as they themselves expressed during the ‘hopes and fears’ discussion. In this context, proposing individual-level actions may have felt more feasible and realistic than suggesting broader environmental interventions that require higher-level resources or structural policy changes. A complementary explanation lies in the composition of the sessions themselves: although facilitation measures were designed to mitigate power imbalances and residents reported feeling heard, proposing environment-changing actions often implies critique of existing policy choices, which may have felt less comfortable for residents with policymakers in the room — and, conversely, policymakers may have been reluctant to propose actions committing resources in front of residents. Separate preparatory sessions per participant group, followed by a joint session, could be considered in future GMB processes.

### Strengths and limitations

A major strength of the study was the direct involvement of adults in socioeconomically disadvantaged situations alongside local policymakers and community stakeholders. This combined lived knowledge of the local environment with knowledge of existing services, institutional constraints and municipal decision-making. Bringing the groups together also created a dynamic of mutual learning where residents gained insight into the institutional considerations behind local decisions, while policymakers were confronted with how their municipality is experienced in daily life. The focus on two peri-urban municipalities also contributes evidence from settings that remain less studied than densely urban environments. Conducting two sessions in each municipality allowed participants to review and revise the municipal CLDs and to return to the process after a period of reflection.

Several limitations should be considered. First, the participant groups were small and purposively selected. Participants in socioeconomically disadvantaged situations had previously participated in CIVISANO research and had expressed an interest in engaging with policymakers. They may therefore have had more confidence, time or social support than other adults experiencing socioeconomic disadvantage. Residents were also numerically outnumbered by professional and institutional participants, and mixed-group power dynamics may have affected what was discussed despite the inclusive facilitation measures used. Related to this, participants in socioeconomically disadvantaged situations contributed primarily from lived experience. Facilitation supported the step from personal experience to general system structure (variables were phrased in generic terms, moderators probed whether mechanisms applied to residents in similar situations), and most participants made this abstraction readily, frequently generalizing to peers and neighbors. Nevertheless, the CLD reflects the range of experiences present in the room, and mechanisms affecting groups not represented may be underrepresented.

Second, the time available limited how deeply participants could explore feedback structures, prioritize actions and assess their feasibility or possible unintended effects. The study did not undertake a formal leverage-point analysis, and the proposed actions should be interpreted as exploratory ideas. Embedding GMB earlier and more iteratively within a research or policy process could create more opportunities to refine, implement and monitor actions.

Third, the identification and naming of subsystems and feedback loops, and the reconciliation of the two municipal models into a merged CLD was produced through an interpretive synthesis by the modelling team. Although it was shared with participants and no comments or requests for changes were received, the absence of feedback should not be interpreted as formal validation.

Finally, a qualitative CLD represents perceived causal structures but does not estimate the strength of relationships, delays, loop dominance or the net effects of interacting feedback processes. A subsequent quantitative system-dynamics model, informed by local data, could examine how these dynamics evolve over time and test how alternative policy combinations might affect the system [73].

## Conclusion

This study shows how adults in socioeconomically disadvantaged situations and local stakeholders perceive unhealthy eating and insufficient walking as outcomes of interacting social, spatial, financial and informational conditions. Six interacting subsystems were identified: 1) social context, 2) facilities and activities, 3) mobility options, 4) affordability, 5) information and 6) marketing and promotion.

Many of the identified mechanisms resemble those found in other obesity-related systems maps, supporting the presence of recurring dynamics across settings. The study adds to this evidence by showing how peri-urban spatial dispersion, limited mobility and social isolation can operate as cross-cutting mechanisms that restrict access not only to food and physical activity opportunities but also to information, services and social connection.

The findings suggest that local action should move beyond isolated information or behavior-change initiatives. Actions that reduce financial, mobility, informational and social barriers simultaneously, and that minimize the effort required from residents to benefit from them, may be particularly valuable. These actions require further co-design, feasibility assessment and evaluation, but they provide a locally grounded basis for developing more integrated and equitable responses.

## Supporting information

S1 Fig De rol van de voedingsomgeving in Duffel. (PDF)

S2 Fig De rol van de wandelomgeving in Duffel. (PDF)

S3 Fig De rol van de beweeg- en eetomgeving in Herselt. (PDF)

S4 Fig CLDs from the first GMB session. (DOCX)

S5 Fig 1^st^ presented CLDs during GMB session 2. (PDF)

S6 Fig Final CLDs GMB session 2. (PDF)
