# Supplementary material for: Understanding the complex systems driving the perceived obesogenic environment among adults in socioeconomically disadvantaged situations: Findings from a participatory Group Model Building process in Flanders
Source: PLoS One. 2026 Oct 5;21(10):e0346991. doi: 10.1371/journal.pone.0346991 (PMC13637927; doi:10.1371/journal.pone.0346991)

De rol van de beweeg- en eetomgeving in Herselt

Uitkomsten van het Civisano-project

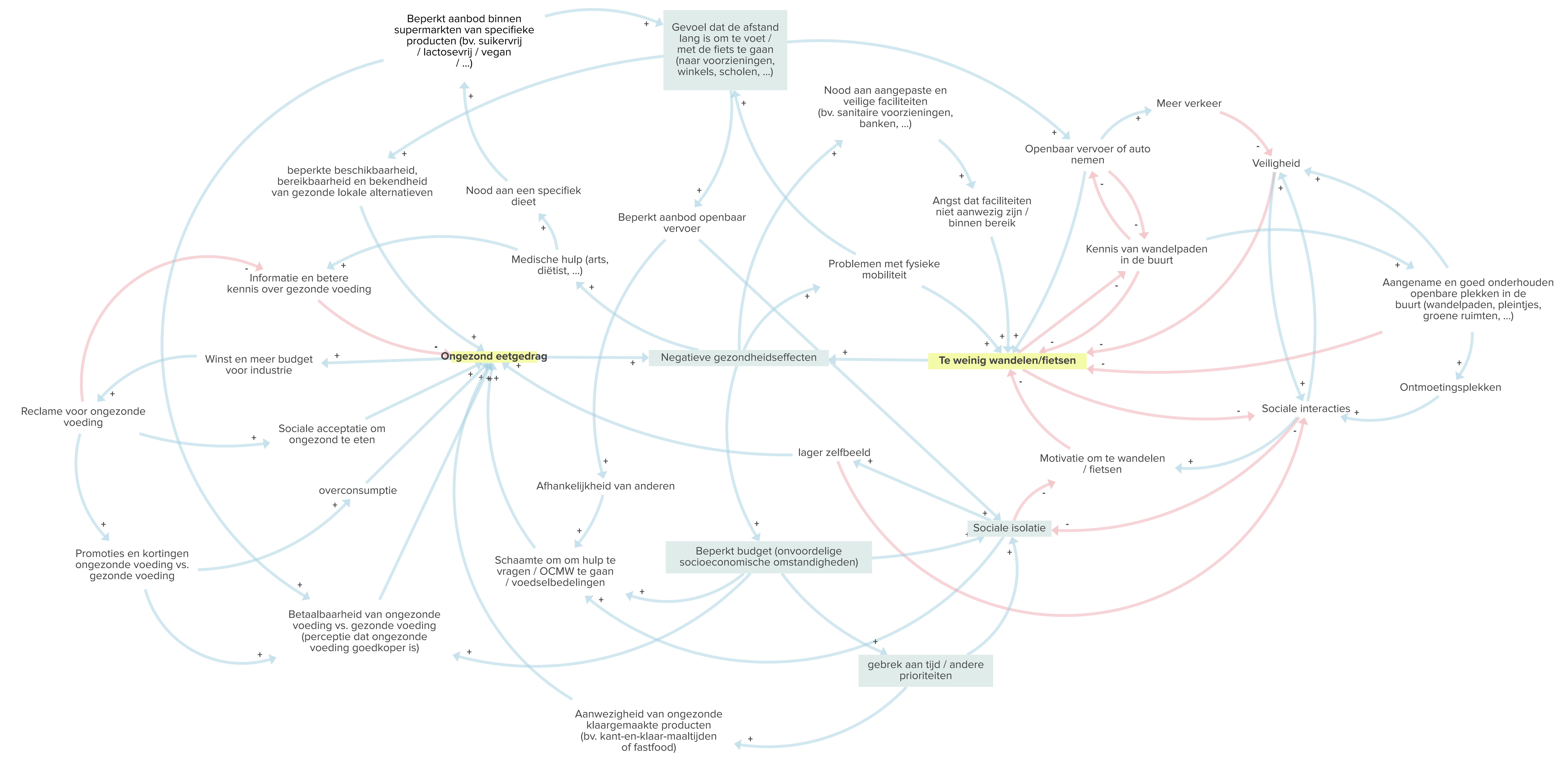

Supplement: Supplementary file 3 [file pone.0346991.s003.pdf]
