# Supplementary material for: Understanding the complex systems driving the perceived obesogenic environment among adults in socioeconomically disadvantaged situations: Findings from a participatory Group Model Building process in Flanders
Source: PLoS One. 2026 Oct 5;21(10):e0346991. doi: 10.1371/journal.pone.0346991 (PMC13637927; doi:10.1371/journal.pone.0346991)

Appendix 5: 1st presented CLDs during GMB session 2

A) CLD - walking behavior - Duffel

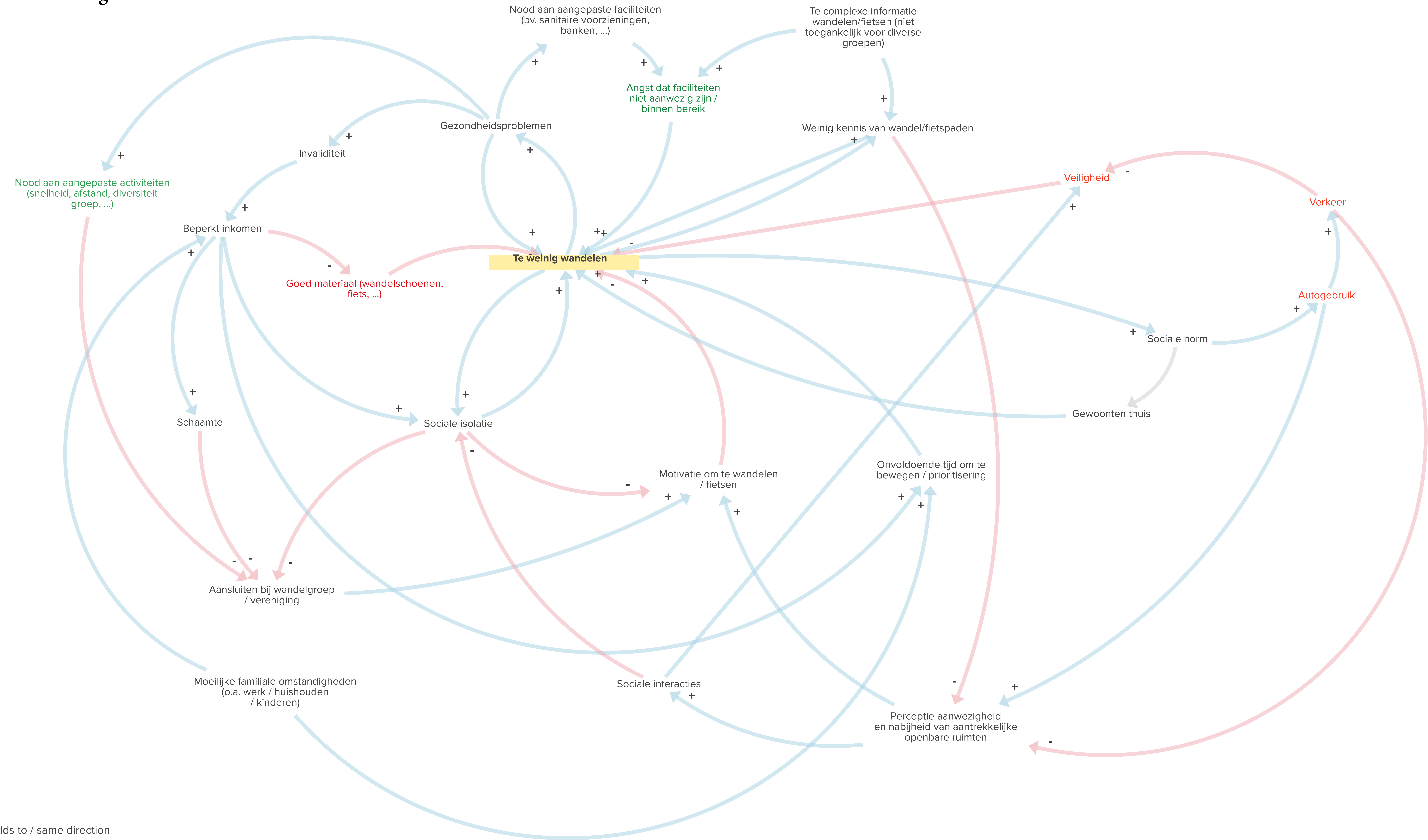

Legend

Adds to / same direction

Subtracts from / opposite direction

B) CLD - eating behavior - Duffel

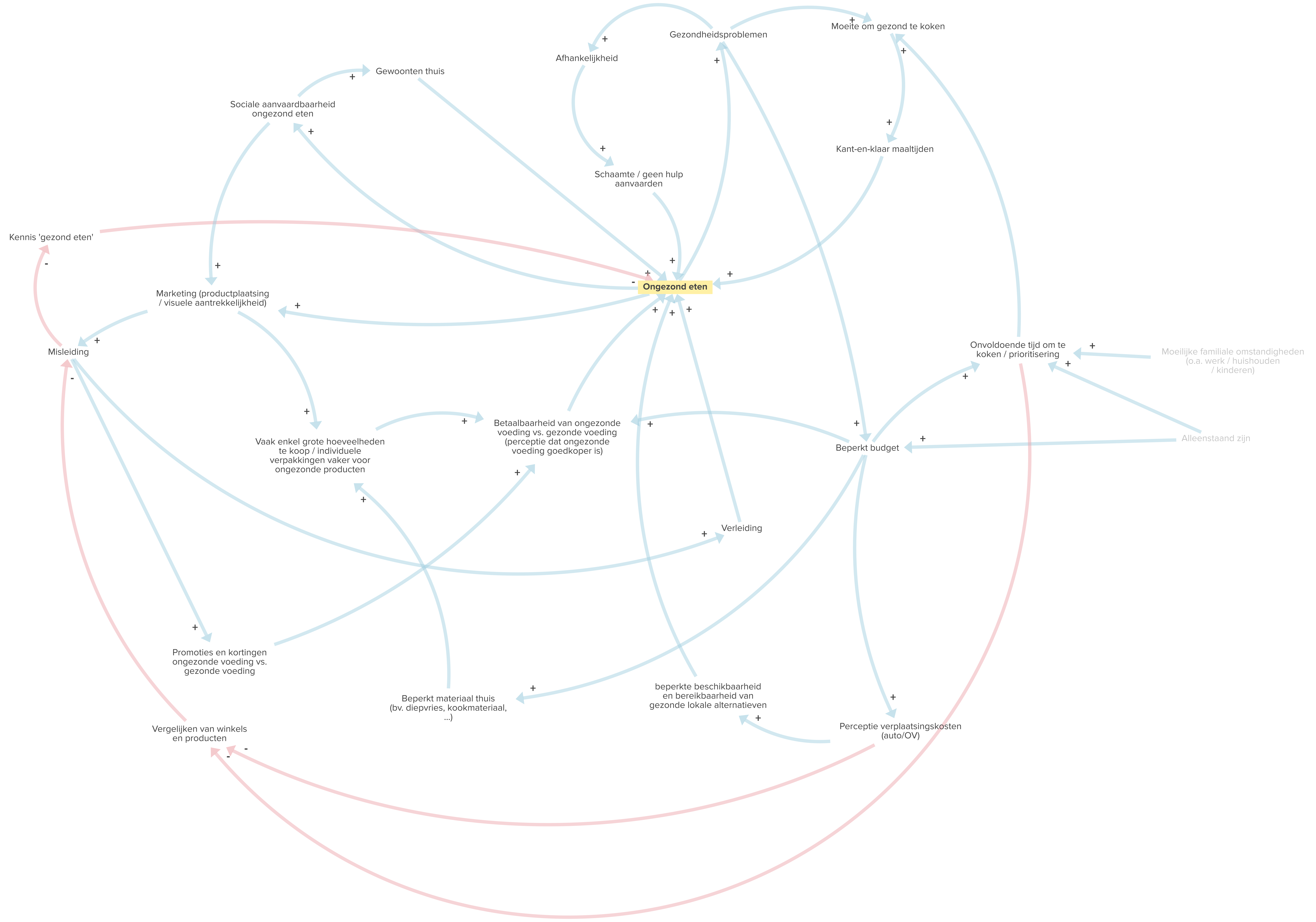

Legend
- Adds to / same direction
- Subtracts from / opposite direction

C) CLD - Herselt

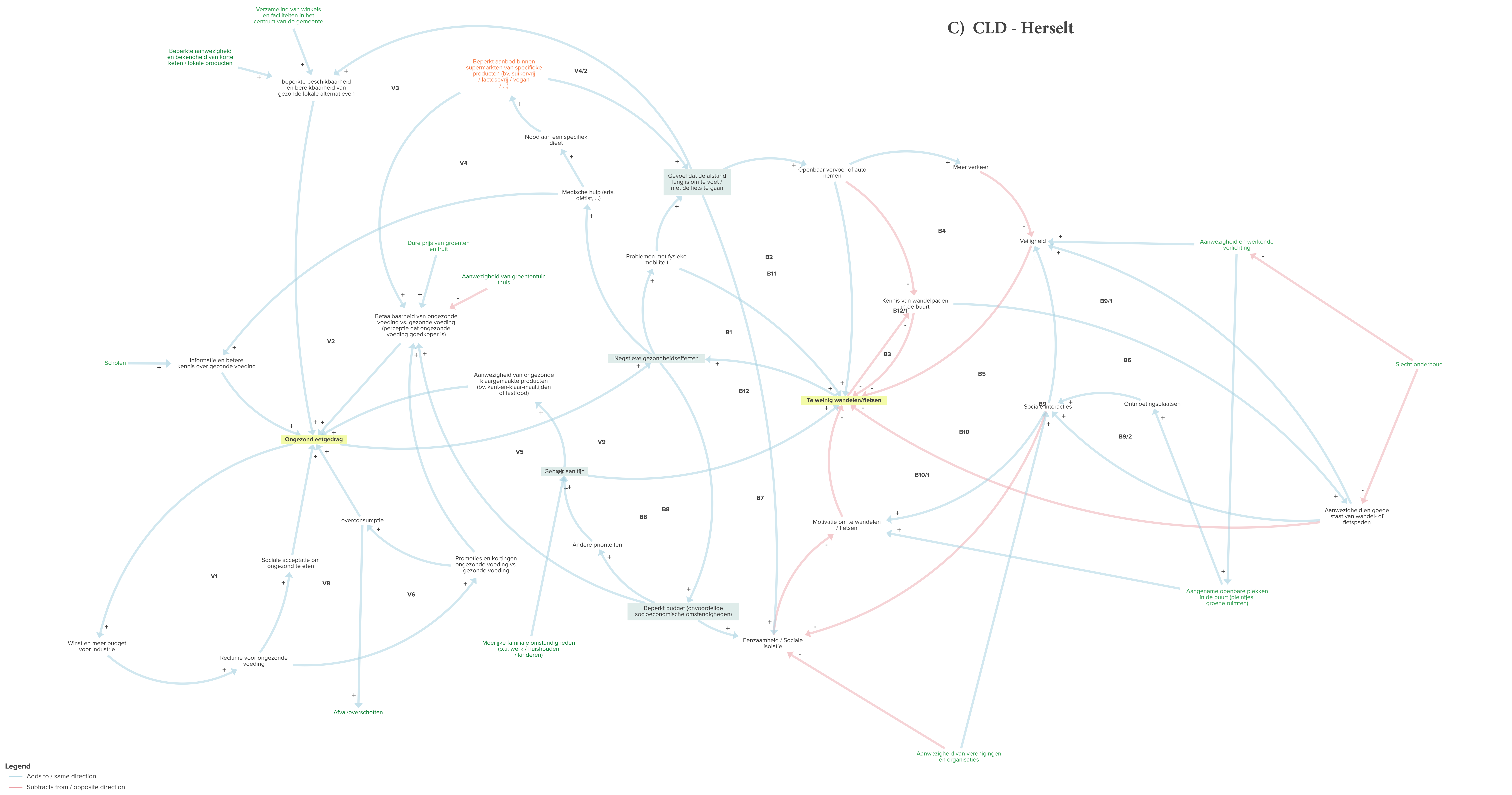

Supplement: Supplementary file 4 [file pone.0346991.s005.pdf]
