# Supplementary material for: Understanding the complex systems driving the perceived obesogenic environment among adults in socioeconomically disadvantaged situations: Findings from a participatory Group Model Building process in Flanders
Source: PLoS One. 2026 Oct 5;21(10):e0346991. doi: 10.1371/journal.pone.0346991 (PMC13637927; doi:10.1371/journal.pone.0346991)
Supplement: Supplementary file 6 [file pone.0346991.s004.docx]

**Appendix 4: CLD’s from the first GMB sessions**

A) **CLD’s in Duffel**

B) **CLD’s in Herselt**
